# Functional connectivity impairment of thalamus-cerebellum-scratching neural circuits in pruritus of chronic spontaneous urticaria

**DOI:** 10.3389/fnins.2022.1026200

**Published:** 2022-10-20

**Authors:** Leixiao Zhang, Zihao Zou, Siyi Yu, Xianjun Xiao, Yunzhou Shi, Wei Cao, Ying Liu, Hui Zheng, Qianhua Zheng, Siyuan Zhou, Junpeng Yao, Yanli Deng, Qian Yang, Sijue Chen, Pingsheng Hao, Ning Li, Ying Li

**Affiliations:** ^1^Department of Integrated Traditional and Western Medicine, West China Hospital, Sichuan University, Chengdu, Sichuan, China; ^2^Acupuncture and Tuina School, Chengdu University of Traditional Chinese Medicine, Chengdu, Sichuan, China; ^3^College of Health Rehabilitation, Chengdu University of Traditional Chinese Medicine, Chengdu, Sichuan, China; ^4^Chinese Medicine Hospital, Chengdu, Sichuan, China; ^5^Sichuan Second Chinese Medicine Hospital, Chengdu, Sichuan, China; ^6^Hospital of Chengdu University of Traditional Chinese Medicine, Chengdu, Sichuan, China

**Keywords:** chronic spontaneous urticaria, pruritus, functional connectivity, thalamus, cerebellum, scratching

## Abstract

Pruritus of chronic spontaneous urticaria (CSU) is one of the most common and irritating sensations that severely affects the quality of life. However, the changes in the functional connectivity (FC) between thalamic subregions and other brain regions have not been fully elucidated. This study aimed to explore the potential changes in brain neural circuits by focusing on various subregions of the thalamus in patients with CSU pruritus to contribute to the understanding of chronic pruritus from the perspective of central mechanisms. A total of 56 patients with CSU and 30 healthy controls (HCs) completed the data analysis. Urticaria Activity Score 7 (UAS7), pruritus visual analog score (VAS-P), Dermatological Life Quality Index (DLQI), and immunoglobulin E (IgE) values were collected to assess clinical symptoms. Seed-based resting-state functional connectivity (rs-FC) analysis was used to assess relevant changes in the neural circuits of the brain. Compared to HCs, seeds within the caudal temporal thalamus (cTtha) on the right side of patients with CSU showed increased rs-FC with the cerebellum anterior lobe (CAL). Seeds within the lateral prefrontal thalamus (lPFtha) on the right side showed increased rs-FC with both CAL and pons, while those within the medial prefrontal thalamus (mPFtha) on the right side showed increased rs-FC with both CAL and the dorsal lateral prefrontal cortex (dlPFC) on the right side. Seeds within the posterior parietal thalamus (PPtha) on the right side showed increased rs-FC with the cerebellum posterior lobe (CPL) on the left side. The UAS7 values and IgE levels were positively correlated with the rs-FC of the right dlPFC. Our results suggest that patients with CSU may exhibit stronger rs-FC alterations between certain thalamic subregions and other brain regions. These changes affect areas of the brain involved in sensorimotor and scratching.

**Trial registration number:**

[http://www.chictr.org.cn], identifier [ChiCTR1900022994].

## Highlights

– Resting-state functional connectivity analysis was used to analyze pruritus of CSU.
– Thalamic subregions were used as seed points for the first time.
– Functional connections with the cerebellum and frontal lobe were found.
– Changes in UAS7 and IgE levels were positively associated with scratching the neural circuitry.
– Functional connectivity disturbances exist in patients with CSU pruritus.

## Introduction

Chronic spontaneous urticaria (CSU) is a global intractable skin disease, defined as urticaria with spontaneous onset in the absence of specific predisposing factors, and characterized by pruritus, wheals, with or without angioedema, with a duration of more than 6 weeks (Kolkhir et al., 2017; Zuberbier et al., 2022). Clinical symptoms more often affect the upper and lower limbs and are aggravated during the summer and at night (Maurer et al., 2010). CSU affects a considerable proportion (1–2%) of the population (Sánchez-Borges et al., 2021). CSU appears to be more prevalent in females than in males (Rosman et al., 2019; Wertenteil et al., 2019) as well as in the geographic locations of Asia than in Europe and North America (Fricke et al., 2020). A hospital-based multicentre epidemiological questionnaire by researchers from China in 2014 found that 61.0% of 3,027 patients were afflicted with CSU (Zhong et al., 2014). The prevalence of CSU increases with increasing disease activity, and more medical resources and costs are required to treat it. The mean annual direct and indirect costs related to CSU in the United States have been estimated to be $244 million, with medication costs accounting for 62.5% and work absenteeism for 15.7% of the expenses (Delong et al., 2008). Owing to the long course of the disease (van der Valk et al., 2002; Toubi et al., 2004; Sánchez-Borges et al., 2017), multiple inducing factors (Zhong et al., 2014), high medical costs, and repetitive nature (Maurer et al., 2017), the physical and mental health, as well as the quality of life of patients, are seriously affected (Balp et al., 2015; Vietri et al., 2015). Symptoms are long-lasting and difficult to control, with pruritus in CSU being the most unbearable symptom, further exacerbated by wheals and angioedema (Zuberbier et al., 2018). Chronic pruritus has received increasing attention as a challenging clinical condition.

In recent years, pruritus research has made great progress in terms of cytokines (Jianli, 2006), pathological morphology (Wang and Ma, 2020), and gene expression (Yunzhou, 2020). The brain is the most advanced center for pruritus regulation. However, how this information is transmitted to the brain as well as the central circuit underlying pruritus-induced scratching behavior, remains largely unclear (Mu et al., 2017; Wang et al., 2018a). The internal neural activity of the brain is non-stimulus-dependent thinking activity while the resting state is considered the most basic spontaneous neural activity of the brain in its original state. Blood oxygen level-dependent functional magnetic resonance imaging (BOLD-fMRI) is a technique used to study brain function indirectly through changes in the ratio of oxygenated to deoxygenated hemoglobin during brain functional activities (Ogawa et al., 1990). BOLD-fMRI has various advantages including non-invasiveness, non-radiation, higher spatial resolution, direct superposition of functional images, precise image positioning, as well as the fact that it can better respond to the most basic spontaneous neural activity of the brain (Crosson et al., 2010). Therefore, resting-state BOLD-fMRI is an application mode suitable for the study of brain mechanisms.

Over the past 15 years, a large number of BOLD-fMRI studies have been conducted on urticaria, atopic dermatitis, nodular prurigo, end-stage renal disease, and psoriasis to reveal the changes in the brain function in patients with these skin diseases (Mueller et al., 2017). The cognitive activities of the brain are not only related to the functional activities of specific brain regions, but also the interaction and connection between spatially distributed brain regions, while the brain network may play a more important role (Li, 2014). Resting-state functional connectivity (rs-FC) refers to the temporal correlation of the activities of different brain regions that are spatially separated (Friston et al., 1993) and is mainly used to elucidate the brain functional network (Crosson et al., 2010). Therefore, rs-FC is the analytical method most commonly used to explain functional brain networks (Friston et al., 1993; Crosson et al., 2010). The thalamus is the higher center of sensation, most often activated by harmful stimuli. It is also the core brain region in the pruritus matrix (Najafi et al., 2021). Previous studies have found that when pruritus occurs, the primary afferent nerve from the skin projects the pruritus signal upward to the thalamus, activating several brain regions, including the premotor area, auxiliary motor area, anterior cingulate gyrus, insula, main somatosensory cortex, and sub-somatosensory cortex, prefrontal cortex, orbitofrontal gyrus, precuneus, and cerebellum (Andrew and Craig, 2001; Mochizuki et al., 2003; Paus et al., 2006; Mochizuki and Kakigi, 2015). Among these, the thalamus is associated with the recognition of pruritus and the intensity thereof (Darsow et al., 2000). The thalamus is composed of various nuclei (Long et al., 2020), of which the main nucleus of each thalamus is known to be associated with one or more cortical regions (Müller et al., 2020). Each subregion of the thalamus can be activated by histamine or non-histamine substances (Najafi et al., 2019). Although the whole thalamus has been proven to be an important brain region for CSU patients to transmit pruritus signals, it is not clear which specific sub-brain region of the thalamus is key and how rs-FC changes.

In this study, we primarily discussed the changes in functional connections between each subregion of the thalamus and other brain regions in patients with CSU and whether the changes in these functional connections are correlated with the clinical symptoms of patients with CSU. This study provides direct evidence for a better understanding of the changes in brain function at CSU.

## Materials and methods

### Participants

We recruited 60 patients with CSU in Chengdu, Sichuan Province, China, from 1 January 2020 to 30 June 2021. Diagnostic criteria were based on “the international EAACI/GA^2^LEN/EuroGuiDerm/APAAACI guidelines for the definition, classification, diagnosis, and management of urticaria” (Zuberbier et al., 2022). A total of 31 healthy controls (HCs) were age- and sex-matched with patients with CSU and were recruited locally through advertisements.

### Inclusion criteria

The inclusion criteria were as follows: (1) meet the diagnostic criteria; (2) right-handed, 18 years old ≤ age ≤ 70 years old, education ≥ 6 years, both male and female; (3) Urticaria Activity Score 7 (UAS7) > 14 scores; (4) no metal implants in the body, and no contraindication to fMRI scanning; (5) not using antihistamines within 2 weeks before entering the study, and not using steroid hormones and immunosuppressive drugs within 1 month; (6) not receiving acupuncture treatment or participating in other ongoing clinical studies 3 months before entering the study; and (7) signed the informed consent form and voluntarily participated in this study. Patients who met the above seven criteria were included in this study.

### Exclusion criteria

The exclusion criteria were as follows: (1) contraindications to MRI examinations such as claustrophobia; (2) inability to understand or record the urticaria diary; (3) pregnant and lactating women; (4) combined with serious primary diseases of the cardiovascular, liver, kidney, digestive, and hematopoietic system; (5) progressive malignant tumor or other serious wasting diseases, easily complicated by infection and bleeding; (6) unconscious, unable to express subjective discomfort symptoms, or mentally ill; and (7) participation in similar studies within 1 month of this study. Patients who met any of the above criteria were excluded.

### Clinical symptoms tests

All patients with CSU and HCs completed a series of clinical symptom questionnaires and physiological and biochemical examinations, including age, sex, and disease course, within 1 day of the MRI data acquisition. The UAS7 was the primary outcome of the study. Immunoglobulin E (IgE), pruritus visual analog score (VAS-P), and Dermatology Life Quality Index (DLQI) were secondary outcomes.

### Magnetic resonance imaging data acquisition

All participants completed brain imaging data acquisition using a GE MR750 3.0T (GE Medical Systems, Waukesha, WI, USA) imaging system in the MRI room of the Affiliated Hospital of Chengdu University of Traditional Chinese Medicine. Structural images were acquired using high-resolution three-dimensional T1-weighted brain volume MRI sequences: repetition time (TR)/echo time (TE) = 2,700 ms/3.39 ms; field of view (FOV) = 256 mm × 256 mm; slice thickness = 1 mm; slice number = 176; matrix size = 256 × 256; and flip angle = 7°. Subsequently, axial functional images were obtained using a gradient-echo T2*-weighted echo planar imaging sequence. The scanning parameters were as follows: TR/TE = 2,000/30 ms; FOV = 240 mm × 240 mm; slice thickness = 4 mm; slice number = 43, matrix size = 64 × 64; and flip angle = 90°. Based on these parameters, 240 volumes were acquired in approximately 10 min. The participants were instructed to refrain from drinking coffee, strong tea, and alcohol for at least 24 h and to maintain adequate sleep. Hair sprays and wax were to not be used on the day of data collection. Participants had to arrive at the examination room at least 30 min in advance. Before entering the examination room, we confirmed the absence of cardiac pacemakers, cochlear implants, metal dentures, or intrauterine devices. During the entire scanning process, participants were asked to keep their head and limbs immobile and to relax and close their eyes to rest, but not to fall asleep.

### Data pre-processing

Blood oxygen level-dependent functional magnetic resonance imaging data were converted into an analyzable NIFTI file format using DICOM 1.3.5 (Digital Imaging and Communications in Medicine) software. Resting state-fMRI (Rs-fMRI) data were pre-processed by DPABI software (Data Processing and Analysis of Brain Imaging)^1^ (Yan et al., 2016). This was based on the SPM12 data analysis toolkit^2^ in MATLAB (MathWorks, Natick, MA, USA). The steps were as follows: (1) removal of the first 10 time points; (2) temporal layer correction; (3) head motion correction; (4) spatial normalization (re-acquisition of 3 mm × 3 mm × 3 mm voxel images); (5) spatial smoothing (using an isotropic Gaussian kernel with a full width at half maximum of 6 mm); (6) removing linear trends; (7) regressing head motion effects (using Friston 24 parameters), white matter, and cerebrospinal fluid signals; (8) filtering noise (0.01–0.08 Hz using bandpass filtering); and (9) exclusion of participants whose head moved more than 2.5 mm on any axis or whose head rotated more than 2.5°. Rs-FC was further calculated after pre-processing the Rs-fMRI data.

Four patients in the CSU group were excluded from fMRI data image analysis, (three patients had axial head movement exceeding 2.5 mm, and one patient had head rotation exceeding the threshold of 2.5°) while one subject in the HCs group was excluded because the axial head movement exceeded 2.5 mm. Ultimately, there were 56 members in the CSU group and 30 in the HCs group for the final data analysis.

### Analysis of rs-FC based on seed

The tool used for rs-FC analysis was the CONN-fMRI Functional Connectivity Toolbox v17.a (Whitfield-Gabrieli and Nieto-Castanon, 2012). Regions of interest (ROI) were the various subregions of the thalamus. The brainnetome atlas of the various subregions of the thalamus, such as ROI names and Montreal Neurological Institute (MNI) coordinate maps, were obtained from the Brainnetome Atlas_BNA_subregions^3^ (Table 1; Fan et al., 2016). Using each ROI of the thalamus as a seed, the rs-FC of patients with CSU (*n* = 56) and HCs (*n* = 30) seeded versus the whole brain at baseline were compared. The average time series of all voxels in the seed were calculated and Pearson correlation calculations with other voxel time series in the brain were performed one by one. For each voxel in the whole brain, the correlation coefficient between the voxel and seed was obtained. Pearson correlation coefficients were converted to approximate Gaussian distributed data values using Fisher Z transform. Brain regions with statistically significant relationships were identified and shown based on specific thresholds, and rs-FC between seeds and these brain regions was thus determined (Greicius et al., 2003; Ruirui, 2019; Yi and Chengxin, 2019).

**TABLE 1:** Regions of interest within the thalamus.

Labels	Regions		Hemisphere	MNI coordinates		
	Full name	Abbreviation		x	y	z
1	Medial prefrontal thalamus	mPFtha	L R	−7 7	−12 −11	5 6
2	Medial premotor thalamus	mPMtha	L R	−18 12	−13 −14	3 1
3	Sensory thalamus	Stha	L R	−18 18	−23 −22	4 3
4	Rostral temporal thalamus	rTtha	L R	−7 3	−14 −13	7 5
5	Posterior parietal thalamus	PPtha	L R	−16 15	−24 −25	6 6
6	Occipital thalamus	Otha	L R	−15 13	−28 −27	4 8
7	Caudal temporal thalamus	cTtha	L R	−12 10	−22 −14	13 14
8	Lateral prefrontal thalamus	lPFtha	L R	−11 13	−14 −16	2 7

The regions were selected from a previous study (Fan et al., 2016). L, left; R, right; MNI, Montreal Neurological Institute.

### Statistical analysis

#### Clinical data

SPSS 22.0 (SPSS Inc., Chicago, IL, USA) was used for statistical analysis. Continuous variables were expressed as the mean ± standard deviation (SD) while binary variables were expressed as percentages. Differences in age, medical history, clinical symptoms, and biochemical characteristics between groups were determined using independent two-sample *t*-tests. Differences in sex between the groups were tested using Pearson’s chi-squared test.

#### Imaging data

Seed-based between-group differences in rs-FC were calculated using the SPM12. Two-sample independent *t*-tests were performed on patients with CSU and HCs at baseline. All fMRI data analyses were performed with a voxel-level *p* < 0.005 uncorrected thresholds and a cluster-level *p* < 0.05 family-wise error (FWE) corrected threshold. Brain regions with significant differences were identified in the above analysis. The mean Fisher Z scores for these regions were then extracted and correlated with the clinical outcomes. The threshold for these correlation analyses was two-tailed (*p* < 0.05).

### Ethics statement

This study was approved by the Sichuan Regional Ethics Review of the Committee of Traditional Chinese Medicine (23 April 2019 approval number 2019 kl-006). This research was conducted in accordance with the Code of Ethics of the World Medical Association (Declaration of Helsinki). Informed consent was obtained from all patients with CSU and healthy participants.

## Results

### Demographic and clinical characteristics

The demographic and clinical information of the 56 patients with CSU and 30 HCs are summarized in Table 2. The two groups were matched for age (41.11 ± 12.62, 43.63 ± 15.65, respectively, *p* = 0.419) and sex (females represented 76.8 and 66.7%, respectively, *p* = 0.312). Since patients with CSU had severe urticaria symptoms, the course of the disease, IgE (*p* = 0.000), UAS7, VAS-P, and DLQI (*p* = 0.000) was significantly higher in patients with CSU than in HCs.

**TABLE 2:** Baseline characteristics of patients with CSU and HCs.

Variables	CSU (*n* = 56)	HCs (*n* = 30)	*P*
Age, mean, year	41.11 ± 12.62	43.63 ± 15.65	0.419*^a^*
Female	43(76.8%)	20(66.7%)	0.312*^b^*
Course of disease, month	84.41 ± 48.12	–	–
IgE	247.73 ± 199.08	60.43 ± 40.50	0.000*^a^
UAS7	28.27 ± 7.40	–	–
VAS-P	5.13 ± 2.03	–	–
DLQI	11.21 ± 5.46	0.23 ± 0.50	0.000*^a^

^*a*^The data were tested using an independent two-sample *t*-test.

^*b*^The data were tested using Pearson’s chi-squared test.

*There was statistical significance between the two groups.

CSU, chronic spontaneous urticaria; HCs, healthy controls; IgE, immunoglobulin E; UAS7, urticaria activity score 7; VAS-P, pruritus visual analogue score; DLQI, Dermatology Life Quality Index.

### Characteristics of rs-FC changes between thalamic subregions and brain regions

#### Enhancement of rs-FC (CSU > HCs)

**FIGURE 1:**
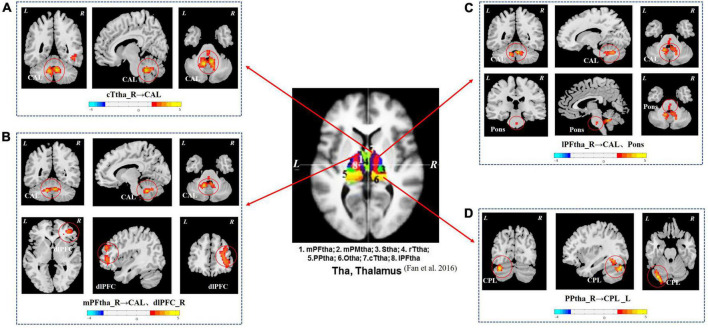
The group differences in thalamus rs-FC network (CSU vs. HCs). **(A)** Seeds within the cTtha_R of patients with CSU showed increased rs-FC with the CAL. **(B)** Seeds within the mPFtha_R showed increased rs-FC with both CAL and dlPFC_R. **(C)** Seeds within the lPFtha_R showed increased rs-FC with both CAL and pons. **(D)** Seeds within the PPtha_R showed increased rs-FC with the CPL_L. Warm colors represent increased rs-FC values. rs-FC, resting-state functional connectivity; CSU, chronic spontaneous urticaria; HCs, healthy controls; L, left; R, right; cTtha, caudal temporal thalamus; mPFtha, medial prefrontal thalamus; lPFtha, lateral prefrontal thalamus; PPtha, posterior parietal thalamus; CAL, cerebellum anterior lobe; dlPFC, dorsal lateral prefrontal cortex; CPL, cerebellum posterior lobe.

As shown in Figure 1 and Table 3, the seed-based rs-FC analysis yielded the following results. When compared to HCs, seeds within the caudal temporal thalamus (cTtha) on the right side of patients with CSU showed increased rs-FC with the cerebellum anterior lobe (CAL). Seeds within the lateral prefrontal thalamus (lPFtha) on the right side showed increased rs-FC with both CAL and pons. Seeds within the medial prefrontal thalamus (mPFtha) on the right side of patients with CSU showed increased rs-FC with both CAL and dorsal lateral prefrontal cortex (dlPFC) on the right side. Seeds within the posterior parietal thalamus (PPtha) on the right side showed increased rs-FC with the cerebellum posterior lobe (CPL) on the left side. No increase in rs-FC was observed in the remaining thalamic subregions and cerebral brain regions in patients with CSU as compared to HCs.

**TABLE 3:** Brain regions with increased seed-based rs-FC values in patients with CSU compared to HCs.

Seed	Contrast	Brain region	BA	Voxel size	MNI coordinates			Peak Z score
					x	y	z	
cTtha_R	CSU > HCs	Cerebellum anterior lobe	–	384	6	−48	33	5.42
	CSU < HCs	None	–	–	–	–	–	–
lPFtha_R	CSU > HCs	Cerebellum anterior lobe	–	250	6	−48	−33	4.51
		Pons	–	39	3	−23	−38	2.77
	CSU < HCs	None	–	–	–	–	–	–
mPFtha_R	CSU > HCs	Cerebellum anterior lobe	–	211	39	42	0	5.39
		dlPFC_R	46/47	211	39	42	0	3.77
	CSU < HCs	None	–	–	–	–	–	–
PPtha_R	CSU > HCs	Cerebellum posterior lobe_L	19	221	−36	−75	−24	3.75
	CSU < HCs	None	–	–	–	–	–	–

CSU, chronic spontaneous urticaria; HCs, healthy controls; rs-FC, resting-state functional connectivity; BA, Brodmann’s area; MNI, Montreal Neurological Institute; R, right; L, left; dlPFC, dorsal lateral prefrontal cortex; cTtha, caudal temporal thalamus; lPFtha, lateral prefrontal thalamus; mPFtha, medial prefrontal thalamus; PPtha, posterior parietal thalamus.

#### Decrease of rs-FC (CSU < HCs)

No decrease in thalamic subregions or cerebral brain regions was found in patients with CSU as compared to HCs.

#### Correlations between seed-based rs-FC and clinical data

**FIGURE 2:**
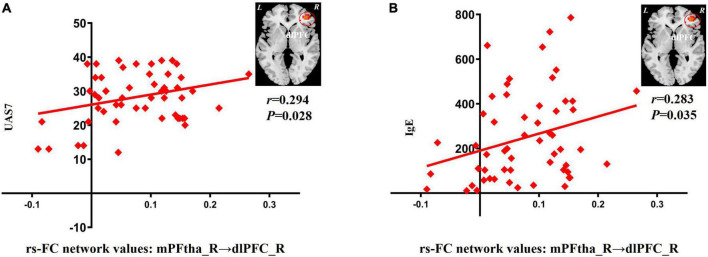
Correlation between increased rs-FC network values and clinical indicators. **(A)** The increase of rs-FC network values between the mPFtha_R and the dlPFC_R was positively correlated with UAS7 (*r* = 0.294; *P* = 0.028). **(B)** The increase of rs-FC network values between the mPFtha_R and the dlPFC_R was positively correlated with IgE (*r* = 0.283; *P* = 0.035). rs-FC, resting-state functional connectivity; L, left; R, right; mPFtha, medial prefrontal thalamus; dlPFC, dorsal lateral prefrontal cortex; UAS7, urticaria activity score 7; IgE, immunoglobulin E.

In patients with CSU, increased rs-FC between the right mPFtha and dlPFC positively correlated with the UAS7 score (*r* = 0.294; *P* = 0.028) and IgE (*r* = 0.283; *P* = 0.035), respectively (Figure 2).

## Discussion

In our study, we applied the voxel level of the seeds to study the alterations in rs-FC between thalamic subregions and cerebral brain regions in patients with CSU. To determine the relationship between these connectivity changes and clinical symptoms, we explored the pathophysiological basis of CSU. In our study, we found an increase in the rs-FC between cTtha on the right and CAL; lPFtha on the right and CAL, pons; mPFtha on the right and CAL, dlPFC on the right; and PPtha on the right and CPL on the left. We did not find, however, any reduction in the differential rs-FC. The mPFtha and dlPFC on the right were positively correlated with UAS7 and IgE, respectively. Since these are pathological features of CSU, these findings provide evidence of rs-FC alterations in the thalamus of patients with CSU.

The thalamus is a key structure in the brain and is considered to be a relay station. It processes all the sensory signals from different parts of the body and relays them to the cerebral cortex. Because of its importance, minimal damage to the thalamus can negatively affect other brain regions (Chen et al., 2021). The thalamus is a complex and diverse brain region comprising many nuclei with diverse physiological functions. The most characteristic feature of CSU is unbearable pruritus (Viegas et al., 2014). Pruritus stimulates pruriceptors, and the afferent fibers transmit impulses to the posterior horn of the spinal cord to realize signal processing at the spinal cord level. Finally, the signal is transmitted to the higher brain center and projected to the corresponding target area through the thalamic nuclei (Wallengren, 2005). The differences in connectivity between different thalamic subregions and other brain regions may be related to the different functions of the thalamic subregions themselves. Pruritus sensation is widely distributed in the frontal and parietal cortices as well as in the subcortical regions in a highly dispersed manner, indicating that the central regulation of pruritus is coordinated by the interaction of multiple functionally related brain regions (Mochizuki and Kakigi, 2015).

In this study, we observed increased connectivity of the thalamus to the prefrontal cortex and cerebellum in patients with CSU. It is important to note that connectivity disturbances in this circuit are primarily located in thalamic regions, such as the cTtha, mPFtha, lPFtha, and PPtha. cTtha, is centered in the dorsal and ventral nuclei and connects with the premotor and somatosensory cortices, as well as parts of the temporal and inferior occipital lobes (Xi et al., 2020). cTtha is considered a first-order nucleus that receives input from the surrounding sensory organs and subcortical structures and sends projections to the motor and somatosensory cortices (Jones, 2007). Based on thalamic histology, mPFtha, and lPFtha are dorsomedial nuclei and anterior complexes that are thought to project into the prefrontal cortex (Russchen et al., 2010). The higher-order nuclei in the thalamus are the mid-dorsal nuclei, which directly receive input from the higher-order associative cortex, including the prefrontal cortex (Jones, 2007). Fan et al. (2016) found that the right PPtha is associated with attention, vision, and perception. Considering that PPtha is related to interoception, this may be related to impaired interoceptive memory function in patients with CSU, which leads to excessive attention to certain feelings, such as pruritus (Li et al., 2019).

Interestingly, aberrant rs-FC between multiple thalamic subregions and the cerebellum was observed in the present study. The cerebellum is a subcortical part of the motor system, much like the thalamus, and is generally thought to be involved in motor coordination (Schneider et al., 2008). The cerebellum may also be involved in sensory coordination as well as cognitive and affective changes (Yosipovitch et al., 2008). In recent years, the role of the cerebellum in patients with CSU has received increased attention. Neuroanatomical studies have shown that the cerebellum sends projections to the sensorimotor and reward areas of the cortex through the thalamus (Schmahmann, 1996). At the same time, the cerebellum receives cortical input from the reward regions *via* the pontine nucleus and from the sensorimotor regions *via* the pontine nucleus and inferior olives. Severe pruritus sensations can prompt repeated scratching. Scratching is a fundamental behavioral response to pruritus that is highly rewarding and relieving (Yosipovitch et al., 2008). Humans are a combination of the body and mind, so pruritus cannot be separated from scratching. What is the effect of scratching? The first is to eliminate pruritus through pain caused by scratching. Second, it is a highly rewarding and seemingly addictive behavioral response (Yosipovitch et al., 2007). The rewarding effect of scratching may be amplified by the presence of pruritus. Thus, scratching can inhibit the emotional component of pruritus, resulting in relief (Habas et al., 2004). One study also found significant activity in the cerebellar hemispheres during pruritus and suggested that these activities were related to the urge to scratch (Herde et al., 2007). The cerebellar efferent pathways from the cerebellum to the reward and sensorimotor areas may be involved in the neuropathology of CSU (Wang et al., 2018b).

When scratching becomes an active action, the role of the sensorimotor cortex is significantly weakened and the cerebellum becomes an important core brain area for processing scratching information (Therrien and Bastian, 2015; Wang et al., 2018b). Voxel-based morphometry (VBM) analysis revealed that patients with CSU showed significantly higher gray matter (GM) volumes in the right premotor cortex, left fusiform cortex, and left cerebellum than HCs (Wang et al., 2021), further demonstrating the important changes in the cerebellum on the pathological basis of CSU. Functional and morphological abnormalities of the cerebellum have been demonstrated in conditions with chronic pruritus, such as psoriasis and CSU. However, the mechanism of pruritus involving the cerebellum is unclear (Wang et al., 2021).

Pruritus and pain showed similar results. Pruritus is considered a multidimensional phenomenon with sensory, emotional, and cognitive aspects similar to chronic pain (Ishiuji et al., 2009). Consequently, pain fMRI studies have found that the dlPFC is an important regulatory area. It exerts active control over pain perception by modulating cortical-cortical and cortical-subcortical interactions (Napadow et al., 2015). The dlPFC and thalamus are major components of the dorsal cognitive circuit and are involved in working memory and executive function (Pauls et al., 2014; Moon and Jeong, 2015; Spiegel et al., 2017). The increased phase of pruritus produces an increase in fMRI signals in the dlPFC compared to saline (Napadow et al., 2015). CSU is caused by degranulation of the mast cells, resulting in excessive histamine release. The fMRI signal of dlPFC was also enhanced upon histamine stimulation (Ishiuji et al., 2009). Peak itchiness is associated with activation in the right dlPFC, bilateral premotor areas, and left superior parietal lobule (SPL) (Napadow et al., 2014). Repeated scratching induced brain activity in the dlPFC, which is consistent with previous imaging studies of pruritus (Mochizuki and Kakigi, 2015). The pons lies between the midbrain and medulla oblongata and is connected to the cerebellar cortex through white matter nerve fibers, transmitting nerve impulses from one cerebellar hemisphere to the other, and coordinating muscle activity on both sides of the body. The pons has been extensively researched in the past for their role in pain sensation. In migraine with aura, intrinsic brain FC between the pons and somatosensory cortex increases during attacks compared to that without attacks (Hougaard et al., 2017).

In patients with CSU, the right mPFtha and dlPFC may play key roles in the pruritus-scratch cycle. Increased rs-FC between the mPFtha and dlPFC on the right was positively correlated with both UAS7 and IgE levels. UAS7 is recommended by the latest chronic urticaria activity guidelines and can be used to assess the number of urticaria and severity of pruritus over 7 days (Zuberbier et al., 2022). Another important pathogenic mechanism of CSU is mediated by the IgE high-affinity receptor (FcεRI) expressed by mast cells (Dobrican et al., 2022). Mast cells are activated upon antigenic stimulation, mediated by IgE, and release multiple mediators, including histamine, to initiate an inflammatory response. A dramatic increase in serum IgE concentration is frequently observed in patients with CSU and is a common indicator of allergic reactions (Tanaka and Furuta, 2021). Most previous CSU functional brain imaging studies have mainly focused on the relationship between clinical symptoms and brain function. However, no attention has been paid to objective changes in IgE levels. To our knowledge, this is the first study to assess the link between these two. Increased rs-FC between the mPFtha and dlPFC on the right side may suggest pruritus-induced sensory hypersensitivity in patients with CSU. The pruritus cycle is the most concentrated manifestation of chronic pruritus and is regulated by multiple brain networks (Wedi, 2022). By comprehensively observing the changes in the clinical symptom score (UAS7) and immune index (IgE), the pathological changes in brain function in patients with CSU can be described in more detail. Objective visual evidence of the pathological mechanism of CSU can be provided.

Previous studies have found that more intense pruritus is associated with the right ventral striatum and right occipital cortex, between the right putamen and left precentral gyrus, and between the precuneus and cingulate cortex rs-FC reduction (Wang et al., 2018a; Dehghan et al., 2022). While it was primarily associated with a decrease in rs-FC, we found that increased connectivity between the SPL and dlPFC was associated with an increase in sensory pruritus. The greater the increase in connectivity, the lower the increase in perceived pruritus, suggesting that greater interactions between nodes in the executive attention network limit pruritus sensation by enhancing top-down regulation (Desbordes et al., 2015). In earlier years, the skin and brain have been shown to be bidirectionally connected, both anatomically and functionally (i.e., skin-brain axis, brain-skin axis) (Arck et al., 2010), as they originate from the common ectoderm (Fan et al., 2016). Under normal circumstances, the hypothalamic-pituitary-adrenal (HPA) axis hormones help maintain skin homeostasis and provide anti-inflammatory and antibacterial defenses (Slominski, 2007; Kim et al., 2013; Chen and Lyga, 2014). Therefore, the HPA axis may change under high stress, leading to skin inflammation and tissue receptor resistance to glucocorticoids (Arck and Paus, 2006; Kim et al., 2013), thereby affecting skin immune homeostasis and inducing or aggravating immune skin diseases (Inanç, 2016). CSU is closely related to visual sensory stimulation. Patients with CSU have frequent wheals and aggravated pruritus, and prolonged visual stimulation of the skin lesions will continue to affect their psychological state. Negative emotions such as tension, depression, and anxiety will further aggravate the pruritus experience (Krishnan and Koo, 2010). The incidence of CSU has seasonal regularity. Most of the patients in this study were admitted during summer and autumn, with a high incidence of disease and aggravation of urticaria or pruritus (Maurer et al., 2010). Other studies have also confirmed that compared with saline, thermal stimulation can increase activation of the anterior auxiliary motor area, dlPFC, and insula, amongst others, in patients with histamine-induced pruritus, which is significantly affected by temperature and has opening-closing properties (Pfab et al., 2008). Therefore, during the onset of clinical symptoms in patients with CSU, the functional connections between several subregions of the thalamus (cTtha, lPFtha, mPFtha, and PPtha) and the cerebellum, pons, and dlPFC were enhanced, and pruritus perception and scratching cycle behaviors were more clearly encoded. Therefore, this may also be one of the reasons for the different results from previous studies.

Our experiment used an rs-FC study. The rs-FC data analysis method based on the seed point method is the simplest and most commonly used method for studying neuronal activity and FC of the brain (Greicius et al., 2003). The rs-FC analysis method observes synchronization between different brain regions from the perspective of functional integration, and studied the presence of a connection between different brain regions and demonstrated the strength of this connection (Horwitz et al., 1992; Yu et al., 2014). Also taking into account the fact that the pons contains the pontine and motor nuclei of the trigeminal and facial nerves and is involved in sensory processes related to touch and pain, facial sensation and expression, and secretion of saliva and tears (Vila-Pueyo et al., 2018), the abnormal connection between the pons and thalamus further confirmed the neural circuit of the pruritus-scratch cycle. To the best of our knowledge, this is the first study to find changes in the function of the pons in patients with CSU.

## Limitations

Although the rs-FC analysis method can intuitively explain the functional changes in the brain, there are also certain subjective and artificial influences on the selection of seed points based on the brain regions accumulated in the past (Teng et al., 2018). Therefore, further research is needed to explore the specific roles of different brain regions in pruritus-related disorders. This study only observed the changes in brain function in patients with urticaria and did not study the brain structure in detail. The two can be combined in the future to further explore the pathological characteristics of urticaria. The primary outcomes identified by fMRI in this study were primarily descriptive where the phenomenon of brain function changes in CSU patients was observed. To understand the mechanism of the phenomena, research protocol will be accordingly changed in the future.

## Conclusion

In this study, we find enhanced rs-FC between the thalamus, cerebellum, and scratching neural circuits in patients with CSU using a seed point-based rs-FC analysis method. Furthermore, abnormally enhanced rs-FC between the right mPFtha and dlPFC was more sensitive to changes in clinical outcomes in patients with CSU. This enhanced rs-FC may contribute to further understanding of the underlying pathological mechanisms of CSU and provide direct evidence for the early diagnosis and recognition of CSU.

## Data availability statement

The raw data supporting the conclusions of this article will be made available by the authors, without undue reservation.

## Ethics statement

The studies involving human participants were reviewed and approved by Ethics Committee of the Hospital of Chengdu University of Traditional Chinese Medicine. The patients/participants provided their written informed consent to participate in this study. Written informed consent was obtained from the individual(s) for the publication of any potentially identifiable images or data included in this article.

## Author contributions

YLi and LZ: conceptualization and funding acquisition. LZ, ZZ, XX, and YLi: project administration. YD, YS, and WC: validation. SY and HZ: formal analysis. YLiu, QZ, and SZ: supervision. JY, QY, and SC: data curation. PH and NL: resources. LZ: writing—original draft. ZZ, SY, and YLi: writing—review and editing. All authors have read this manuscript and approved the publication of the final manuscript.

## Footnotes

1
http://rfmri.org/dpabi

2
https://www.fil.ion.ucl.ac.uk/spm/

3
http://atlas.brainnetome.org/
